# *Salvia officinalis*, Rosmarinic and Caffeic Acids Attenuate Neuropathic Pain and Improve Function Recovery after Sciatic Nerve Chronic Constriction in Mice

**DOI:** 10.1155/2019/1702378

**Published:** 2019-06-24

**Authors:** Zineb El Gabbas, Kenza Bezza, Jawad Laadraoui, Mehdi Ait Laaradia, Aaziz Kebbou, Sara Oufquir, Abderrahman Boukhira, Rachida Aboufatima, Abderrahman Chait

**Affiliations:** ^1^Laboratory of Pharmacology, Neurobiology and Behavior, Semlalia Faculty of Sciences, Cadi Ayyad University, Marrakech, Morocco; ^2^Biochemistry and Toxicology Department, Avicenna Military Hospital, Medical School Faculty, Cadi Ayyad University, Marrakech, Morocco; ^3^Laboratory of Génie Biologique, Sultan Moulay Slimane University, Faculty of Sciences and Techniques, Beni Mellal, Morocco

## Abstract

The leaves of* Salvia officinalis L*. have a traditional reputation for the management of pain in Morocco. This study was conducted to investigate the curative effects of* Salvia officinalis* (SO) and its major constituents Rosmarinic (ROS) and Caffeic acids (CAF) on peripheral neuropathic pain in mice. Chronic constriction injury (CCI) was induced in mice, and neuropathic pain behaviors tests were evaluated by mechanical, chemical, thermal sensation tests and functional recovery of the sciatic nerve at different time intervals,* i.e*., (day 0, 1, 7, 14, and 21). Ethanolic extract of SO (100 and 200 mg/kg,* p.o.*), ROS (10 and 20 mg/kg,* i.p.*), CAF (30 and 40 mg/kg,* i.p.*), and CLOM (5 mg/kg,* i.p.*, a positive control) was given for 21 days after surgery. Hematological and biochemical parameters were also measured as well as histopathological analysis. CCI produced significant development in mechanical and thermal hyperalgesia, cold allodynia, and rise in the sciatic functional index in mice. Chronic treatments with SO extract, ROS, CAF, and CLOM for 3 weeks significantly increased mechanical sensibility, cold, and thermal withdrawal latency and enhanced functional recovery of the injured nerve. The same treatments remarkably ameliorated hematological parameters and did not alter biochemical levels. The histopathological findings had revealed the protective effect of SO, ROS, and CAF against the CCI-induced damage. Our data support the use of SO in folk medicine to alleviate pain. Their main phenolic constituents could be promising antineuropathic compounds, which may be attributed to their biological activities including anti-inflammatory, antioxidant, and neuroprotective effects. SO leaves may be a good candidate to treat neuropathic pain.

## 1. Introduction

Neuropathic pain is a chronic pain caused by injury of somatosensory neurons, which gives rise to pain symptoms that persist over time [1]. It is classified according to three features: the underlying disease, the site of lesion (*i.e*., a peripheral nerve lesion or spinal cord), and the underlying mechanism.

Generally, in neuropathic pain, the symptoms are frequently associated with abnormal sensations and may also be caused by stimuli that can be nonnociceptive (allodynia) or weakly nociceptive, with an amplified reaction (hyperalgesia) [2]. The pathogenesis of neuropathic pain is characterized by neuroinflammation induced activation of immune cells and increased production of several inflammatory mediators, predominantly tumor necrosis factor-*α* (TNF-*α*), interleukin-1*β* (IL-1*β*), and interleukin-33 (IL-33) [3–5], which play an important part in the development of neuropathic pain [6–8]. In addition, epidemiological studies have reported a positive correlation between serum levels of TNF-*α*, C reactive protein (CRP), and erythrocyte sedimentation rate (ESR) in patients with neuropathy [9, 10]. There are also no experimental studies that have examined any correlation between CRP and ESR in animal models of peripheral neuropathy. Therefore, the evaluation of hematological parameters and the concentration of serum CRP could be useful in the diagnosis of neuropathy.

The pharmacological treatments used against this disease are based on the combination of various molecules. Moreover, the available therapies that have been shown to be effective in the treatment of neuropathic pain include antidepressants and antiepileptic drugs. Till this date despite the progress achieved in understanding the pathophysiology of this pain, the treatment remains difficult due to the nonresponse to conventional drug administration and various adverse reactions that lead to poor compliance with treatment [11], which make them unable to attenuate the neuropathic pain [12]. Recently, several scientific reports have demonstrated that herbal plants could be used as an alternative in the treatment of painful neuropathy [13, 14]. One such herb with possible therapeutic utility is* Salvia officinalis* (Lamiaceae family) popularly known as “Salmiya” in Morocco. This plant has been widely used in folk medicine for treating ulcer, diarrhea, gout, rheumatism, tremor, paralysis, and hyperglycemia [15].

A wide range of therapeutic properties as antioxidant [16], anticancer, antimutagenic [9], and antidementia [17] have been revealed. Recently, we have reported the beneficial effect of* Salvia officinalis* (SO) on depression, anxiety, and learning in rats [18]. Besides this, it has been reported that SO also exhibits anti-inflammatory and analgesic activities [19].

Previous phytochemical studies revealed that the main components of SO leaves are flavonoids, (in particular Rosmarinic acid) and the phenolic acids (such as Caffeic acid) [20, 21], which are reported to have a variety of pharmacological activities and potential applications [22–25]. Recently, two reports have evaluated the antineuropathic effect of Rosmarinic acid in CCI rats [8, 26]. More so,* in vivo *investigation of SO extract in mice showed anti-inflammatory effects on Vincristine model [27]. However, so far, there has been no direct evidence regarding the effect of chronic treatment with SO on neuropathic pain induced by CCI model. Additionally, the information on the effect of SO and its major compounds (Rosmarinic and Caffeic acids) on functional recovery and peripheral nerve regeneration in CCI model has been lacking. Therefore, the aim of the present study is to investigate the potential curative effect of* Salvia officinalis* for 3 weeks on hyperalgesia, allodynia and functional recovery of sciatic nerve following the induction of peripheral neuropathic pain in mice through behavioral, biochemical, and histological studies. Clomipramine, a tricyclic antidepressant (TCA), served as a positive control in this study.

## 2. Materials and Methods

### 2.1. Animals

The experiments were performed using Swiss male mice between 24 and 29 g, bred in the central animal care facilities of Cadi Ayyad University, Marrakech, Morocco. Animals were housed (six per cage) under controlled temperature (20±2°C), humidity (60%), and lighting (12/12h light/dark cycle, dark from 7 P.M.), with free access to food and water. All the experimental protocols were carried out in accordance with the European Council Directive (EEC, 1986/609) and duly approved by the Council Committee of research laboratories of the Faculty of sciences, Cadi Ayyad University of Marrakech. All efforts were made to minimize animal suffering and to reduce the number of animals used.

### 2.2. Plant Material

The leaves of* Salvia officinalis *were collected from the Moroccan high Atlas Mountain (Ourika region), in February 2017, and were identified by Professor Ouhammou at the Faculty of Sciences Semlalia, University Cadi Ayyad Marrakech, Morocco. A voucher sample (MARK-10004) has been deposited at the herbarium of the faculty.

The leaves were dried in the shade at 40°C and ground into a fine powder.

### 2.3. Preparation of Ethanolic Extract

The powdered leaves of SO (200g) were extracted with ethanol (750 ml, 80% v/v) in a Soxhlet apparatus for 75h at 65°C. After extraction, the solvent was evaporated by a Rotavapor apparatus at 45°C. The final weight of the extract was 40.2 g.

### 2.4. Phytochemical Analysis of Ethanolic Extract of Salvia officinalis

#### 2.4.1. Determination of Total Phenolic (TPC), Flavonoids (TFC), and Condensed Tannin Contents (TTC)

The total phenolic content of SO ethanolic extract was assessed using Folin-Ciocalteu method [28]. 100 *μ*l of the extract was diluted with 3.7 ml of distilled water, and 200 *μ*l of Folin-Ciocalteu reagent was added. Thereafter, 3 min, 20% sodium carbonate (1 ml, Na2CO3) was added. The solution was agitated and incubated in dark for 45 min at 25°C. Then, absorbance was read at 725 nm a UV-Vis spectrophotometer (VR-2000, Spain). The total phenolic concentration of SO ethanolic extract is reported in terms of mg equivalent Gallic acid per g of the dry matter of extract (DM). All samples were carried out in triplicate.

The flavonoids content of SO extract was determined based on the aluminum trichloride method [29]. Aliquots (200 *μ*l) of diluted extract were added to 5% NaNO2 solution (60 *μ*l), followed by 10% AlCl3 (40 *μ*l), and incubated for 6 min, followed by the addition of NaOH (400 *μ*l). Then, distilled water (500 *μ*l) was added. The absorbance of the solution was read at 510 nm. The TFC in SO was reported as mg equivalent catechin per g of the DM.

Tannins in SO extract are evaluated as follows [30]. Sample extract (300 *μ*l) was added to a solution of vanillin (3 ml, 4% methanol) and HCl (1 ml). The absorbance was measured at 500nm. The results were reported as catechin per g of the extract.

#### 2.4.2. In vitro Antioxidant Activity

*DPPH Assay. *The radical scavenging activity of SO sample was performed by the method of Burda and Oleszek [31]. 25 *μ*l of the different sample or standard concentrations (butylated hydroxyl toluene (BHT) and Quercetin) was added to 2.8 ml of the methanol solution of DPPH (0.004%) and left to stand 60 min at room temperature. The absorbance was recorded at 517 nm. The measurements were carried out in triplicate. The antiradical activity of SO was evaluated as a percentage of DPPH discoloration (IC50 value).

*Reducing Power Assay (FRAP). *The FRAP assay of SO extract was realized according to the method of Oyaizu [32]. The plant (1 ml) was added to the sodium phosphate buffer solution (0.2 M; pH 6.6; 2.5 ml) and 1% of potassium ferricyanide (K3Fe (CN) 6; 2.5ml). After 20 min of incubation at 50°C the solution of Cl_3_CCOOH (2ml) was added and centrifuged (3000×*g*; 10min). Finally, a 2.5 ml aliquot of the mixture was combined with 0.5 ml ferric chloride (0.1%, FeCl3) and 2.5 of water.

Reading absorbance was set at 700 nm. BHT and Quercetin were used as a positive control.

*β-Carotene/Linoleic Acid Bleaching Method*. In this test, the antioxidant capacity of SO ethanolic extract is performed following the method of Kartal et* al*. [33]. *β*-carotene/linoleic acid emulsion was prepared by solubilization of 0.5 mg of *β* carotene in chloroform (1 ml); linoleic acid (25 *μ*l); and 200 mg of Tween 20, then 100 ml of water was added to the solution. The emulsion (2.5 ml) was added to the extract solution (350 *μ*l) or synthetic antioxidant reference (BHT). The absorbance of the prepared emulsion was measured at 470nm.

(1) $$\begin{array}{c} \%\;Inhibition=\left[\;\frac{\left(A_{A\left(2h\right)}\text{-}A_{B\left(2h\right)}\right)}{\text{(}A_{B\left(0\right)}\text{-}A_{B\left(2h\right)}}\;\right]\times 100 \end{array}$$

where A_A(2h)_ and A_B(2h)_ are the absorbances of the sample and control, respectively, after 2 h and A_B(0)_ is the absorbance of control t=0.

#### 2.4.3. Determination of the Polyphenolic Content by HPLC

The high-performance liquid chromatography (Knauer) equipped with a (K-1001) pump and a PDA detector (200-700 UV-Vis) was used to separate and characterize phenolic compounds present in SO extract. The analysis was performed with a Eurospher II 100-5, column (4,6×250 mm), and the temperature was set at 25°C. The flow rate was 1 ml/min, the sample volume injected was 2ml, and the wavelengths of detection were set at 280 nm. Acidified water (A) and acetonitrile (B) mixture were used as the optimal mobile phase for a total running time of 60 min. The identification of phenolic compounds was made by comparison of retention time and spectra with those of commercially available standard compounds.

### 2.5. Induction of Neuropathic Pain by CCI

Neuropathy was induced in animals following the method of Bennett and Xie [34]. The mice were anesthetized by chloral hydrate (300 mg/Kg,* i.p.*). Then four loose ligatures (chromic gut 4-0, with 1 mm spacing) were tied around the left sciatic nerve. Sham-operated mice underwent the same surgical procedure except for the sciatic nerve ligation.

### 2.6. Drugs and Pharmacological Treatments

The following drugs were used: Clomipramine (5 mg/Kg) (Novartis, Maroc, S.A.), Rosmarinic acid 96%, and Caffeic acid purchased from Sigma-Aldrich (St. Louis, MO, USA) and* Salvia officinalis *ethanolic extract (100 and 200 mg/Kg). Drugs were dissolved in 0.9% NaCl solution before administration.

### 2.7. Experimental Design

Animals were assigned into the following 9 groups (n = 6, for each group):

(i) Vehicle group: intact animals treated with normal saline (5 ml/Kg* i.p*.).

(ii) Sham group: mice which underwent the surgical procedure without nerve ligation and treated with normal saline (5 ml/kg* i.p.*).

(iii) SO group: animals which were treated with sage extract* per se* (100 and 200 mg/kg* p.o.*).

(iv) CLOM group: animals which received Clomipramine* per se* (5 mg/kg* i.p.*).

(v) ROS group: animals treated with Rosmarinic* per se* (10 and 20 mg/kg* i.p.*)

(vi) CAF group: animals treated with Caffeic acid* per se* (30 and 40 mg/kg* i.p.*).

(vii) CCI group: animals with chronic constriction of the sciatic nerve.

(viii) CCI+ SO group: CCI animals treated with sage extract (100 and 200 mg/kg* p.o*).

(ix) CCI+ ROS or CAF acid: CCI animals which received Rosmarinic (10 and 20 mg/kg* i.p.*) or Caffeic acid (30 and 40 mg/kg* i.p.*).

(x) CCI+ CLOM group: CCI animals treated with Clomipramine (5 mg/kg* i.p.*).

**Figure 1:**
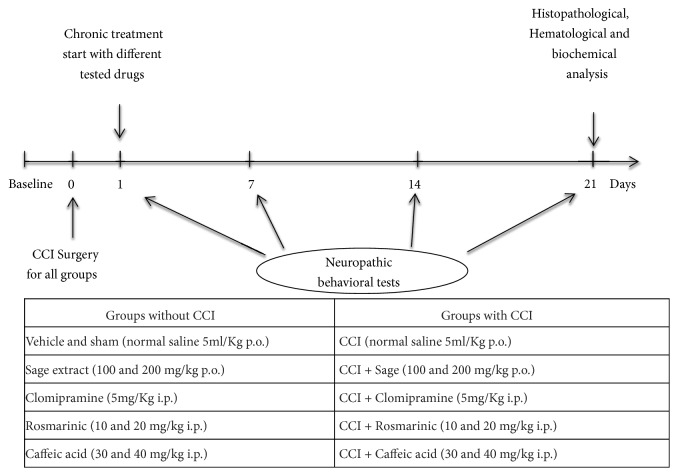
Schematic timeline and experimental design used in this study.* Salvia officinalis* (SO), Rosmarinic acid (ROS), Caffeic acid (CAF), and Clomipramine (CLOM).

As outlined in Figure 1, the experimental protocol followed in this study was initiated (after surgery) by the measurement of baseline behavioral neuropathic pain tests for all tested animals. Mice were divided into 9 groups, and data were collected from six individual mice in each treatment group (n = 6). Then tests were done between 10h and 14h on the 1st day, 7, 14, and 21 after surgery and the behavioral parameters were measured at 40 minutes after chronic treatment with different drugs. At the end of 3 weeks of tests, biochemical, hematological, and histopathological analyses were evaluated.

*Pain Behavior Testing. *Neuropathic pain behavior tests were made up of four tests: mechanical, cold allodynia, thermal hyperalgesia, and sciatic nerve function (walking test).

#### 2.7.1. Mechanical Allodynia (von Frey Test)

In order to evaluate the sensory function of the sciatic nerve, paw withdrawal threshold in the response to mechanical stimuli was measured using von Frey test (Bioseb, USA); 10 filaments, with approximately equal logarithmic incremental bending forces, were chosen [35]. The mice were placed in a plastic cage on a metal mesh floor and were allowed to get used to the environment prior to testing for 10 min. A series of von Frey filaments stimuli with an ascending order of forces, 0,04 g, 0,07 g, 0,16 g, 0,4 g, 0,6 g, 1 g, 1,4 g, 2 g, and 4 g, were applied vertically to the plantar surface of the hind paw. Each filament was tested five times per paw and the mechanical threshold was defined when the tested mice showed at least three withdrawals out of five consecutive trials.

#### 2.7.2. Cold Allodynia (Acetone Test)

For cold allodynia, paw withdrawal latency to cold stimulus was evaluated using the acetone spray test, in which 50 *μ*L of acetone was applied to the plantar surface of mice left hind paw 5 times (at 5 min intervals). For 30 seconds, the mouse cold chemical sensitive reaction was recorded if the mouse lifted the hind paw. The duration of paw withdrawal was measured and expressed as cumulative reaction time [36].

#### 2.7.3. Thermal Hyperalgesia (Hot-Plate Test)

In the hot-plate test, the heat thermal sensitivity of the hind paw was assessed by using hot-plate apparatus (UgoBasil, Italy; Socrel DS-37). In this experiment, the animals were placed on the top of a preheated (52,5°  ± 0,5°C) plate surface, and the time of paw withdrawal reaction was recorded (cut-off time was set at 20 s) [37].

#### 2.7.4. Sciatic Nerve Function (SFI)-Walking Test

In this experiment, SFI was evaluated, as described by De Medinaceli [38], and based on the measurements recorded during walking tracks of tested animals [39].

Walking track analysis was performed on all mice one day before surgery and on days 1, 7, 14, and 21 following surgery.

Prior to the injury, the mice were trained to traverse along a confined walkway 7.5 cm wide by 60 cm, covered with precut white paper, terminating in a cage similar to their home cage, with bedding. Footprints were obtained by dipping both hind paws in a nontoxic finger paint.

To evaluate the degree of the sciatic nerve function, three specific factors were calculated for each of the 3 print measurements and using the following formula [38]:

(2) SFI=−38.3EPL-NPLNPL+109.5ETS-NTSNTS+13.3EIT-NITNIT−8.8

where N is normal, E is experimental, PL denotes print length, TS denotes total toe spreading, and IT denotes the distance from the second to the fourth toe.

The SFI was analyzed as SFI oscillating around 0 considered to reflect normal function;

SFI= - 100 indicates significant impairment.

### 2.8. Hematological and Biochemical Analysis

After 21 days of surgery and behavioral evaluation, blood samples were withdrawn from the orbital sinus of mice for the evaluation of hematological factors:

White blood cells (WBC), neutrophils (NEUT), eosinophils (EO), basophils (BASO), lymphocytes (LYMPH), and total monocytes (MONO). Red blood cell count (RBC), hemoglobin (Hb), hematocrit (HCT), mean corpuscular volume (MCV), mean corpuscular hemoglobin (MCH), mean corpuscular hemoglobin concentration (MCHC), platelets, and erythrocyte sedimentation rate (ESR) were determined using hematology automat XT-4000i.

Moreover, the biochemical assessment of serum CRP level was performed as a biomarker of inflammation.

### 2.9. Nephrotoxicity and Hepatotoxicity Analysis

Urea, Creatinine, Aspartate Aminotransferase (AST), and Alanine Aminotransferase (ALT) were used as indicators of nephrotoxicity [40] and hepatotoxicity [41], respectively.

### 2.10. Histopathological Study

According to Sudoh et* al*. [42], sciatic nerve samples were fixed in (10% of formalin solution), transected (4 *μ*m, thicknesses) and stained (Hematoxylin and Eosin). The qualitative analysis of nerve sections was done under a light microscope (450×) for axonal degeneration.

### 2.11. Statistical Analysis

All the results were expressed as the mean ± standard errors of mean (SEM). Comparison between different groups was made using the one-way analysis (ANOVA) and repeated measure model of ANOVA followed by Tukey's post hoc test. A value of p<0.05 was considered to be statistically significant.

## 3. Results

### 3.1. Phytochemical Analysis of Ethanolic Extract of* Salvia officinalis*

#### 3.1.1. Determination of Total Polyphenols, Flavonoids, and Condensed Tannins Contents Quantification

In the present study, the Salvia officinalis ethanolic extract shows a high level of phenolic compounds (120.30 ± 0.72 mg GAE/g DM), flavonoids (91.12 ± 0.50 mg QEeq/1 g DM), and condensed tannins (22.10 ± 0.41 mg CAT/ g DM) (Table 1).

#### 3.1.2. Antioxidant Activity

Table 2 presents the total antioxidant capacity obtained through the DPPH, reducing power and *β*-carotene assay for ethanolic extract of SO in comparison with that of Quercetin and of BHT (synthetic antioxidant). SO exhibited the best performance in reducing power systems and *β*-carotene/linoleic acid bleaching assay with IC_50_ values of 0.221 ± 0.006 mg/ml and 0.300 ± 0.002 mg/ml, respectively, whereas SO displayed a great antioxidant activity in DPPH free radical scavenging test system (IC_50_ = 0.661 ± 0.021 *μ*g /ml).

#### 3.1.3. Determination of the Polyphenolic Content by HPLC

**Figure 2:**
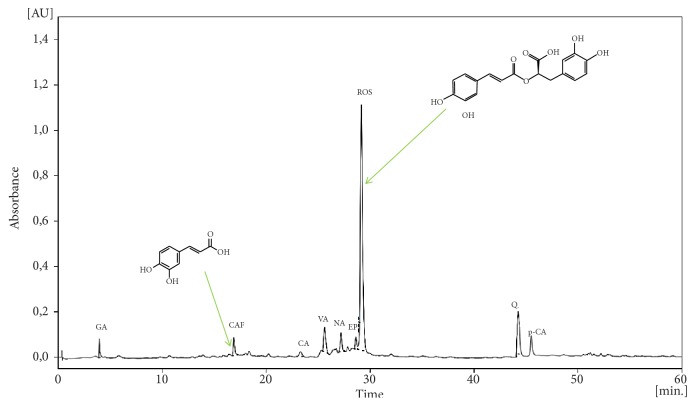
HPLC chromatogram of SO at 280 overlaid. GA: Gallic acid; CAF: Caffeic acid; CA: Carnosic acid; VA: Vanillic acid; NA: Naringenin; EP: Epicatechin; ROS: Rosmarinic acid; Q: Quercetin; p-CA: p-coumaric acid.

HPLC chromatogram analysis indicates the presence of phenolic components in the SO extract namely the Rosmarinic acid as the major phenolic, Caffeic acid, p-coumaric acid, Quercetin, Gallic acid, Vanillic acid, Naringenin, Epicatechin, and Carnosic acid (Figure 2).

The concentrations of phenolic compounds identified in SO are tabulated in Table 3. The phenolic contents in the investigated extract ranged from to 13.64 mg 76.70 mg. Rosmarinic acid is the most abundant compound with the highest concentration (76.70 mg EGA/100 g DM), followed by Quercetin (28.23 mg EGA/100 g DM) and Vanillic acid (17.96 mg EGA/100 g DM).

### 3.2. Behavioral Tests

Sham animals were not statistically different from vehicle animals (data not shown). Animals that were treated for 21 days with SO extract per se (100 and 200 mg/kg), Rosmarinic (10 and 20 mg/kg) and Caffeic acids (30 and 40 mg/kg), and Clomipramine (5 mg/Kg) were also the same as sham animals regarding the behavioral parameters of neuropathic pain.

#### 3.2.1. Effect of* Salvia officinalis* on Mechanical Allodynia Test

A series of von Frey filaments were used to assess the recovery of hypersensitivity to innocuous mechanical stimuli.

**Figure 3:**
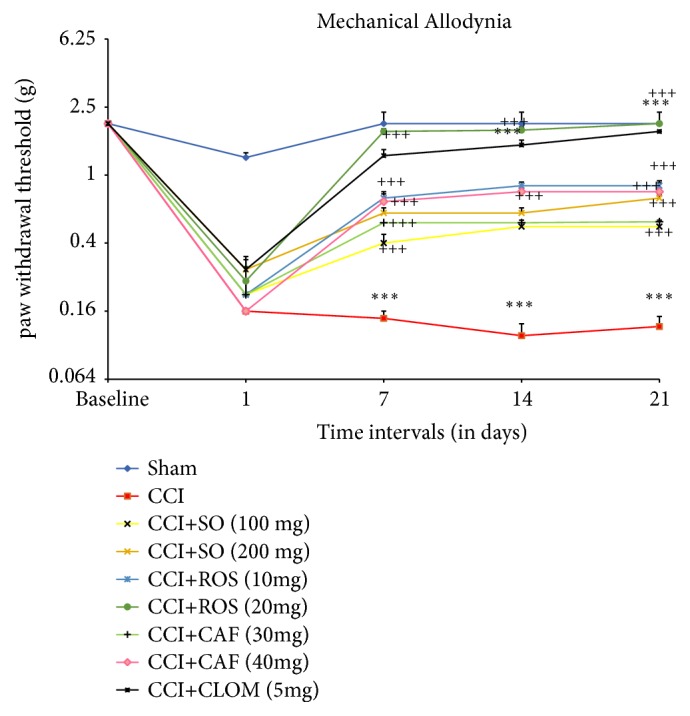
Effect of the administration of* Salvia officinalis *(SO), Rosmarinic acid (ROS), Caffeic acid (CAF), and Clomipramine (CLOM), for 3 weeks, on paw withdrawal threshold CCI of the sciatic nerve in mice (n = 6). SO was orally given in doses of 100 mg/kg and 200 mg/kg. The data are expressed as mean ± SEM. *∗∗∗*p<0.001 compared with sham group; ^+++^p<0.001 compared with CCI group.

At 7, 14, 21 days after repeated oral treatment with SO (100 and 200 mg/kg, as a daily single dose for 21 days), the mechanical withdrawal thresholds in SO group were significantly higher than CCI group (p< 0.001). In addition, mechanical allodynia was significantly attenuated by Rosmarinic acid (10 and 20 mg/kg) and Caffeic acids (30 and 40 mg/kg) during 3 weeks after treatment as analyzed by one-way ANOVA followed by Tukey's multiple comparison test (p< 0.001, Figure 3). All those findings suggest that SO Rosmarinic and Caffeic acids promote mechanical sensation recovery in a dose-dependent manner.

Treatment with Clomipramine at 5 mg/kg also produced similar effects at all assessment time* i.e*., on days 7, 14, and 21.

#### 3.2.2. Effect of* Salvia officinalis* on Cold Allodynia (Acetone Test)

**Figure 4:**
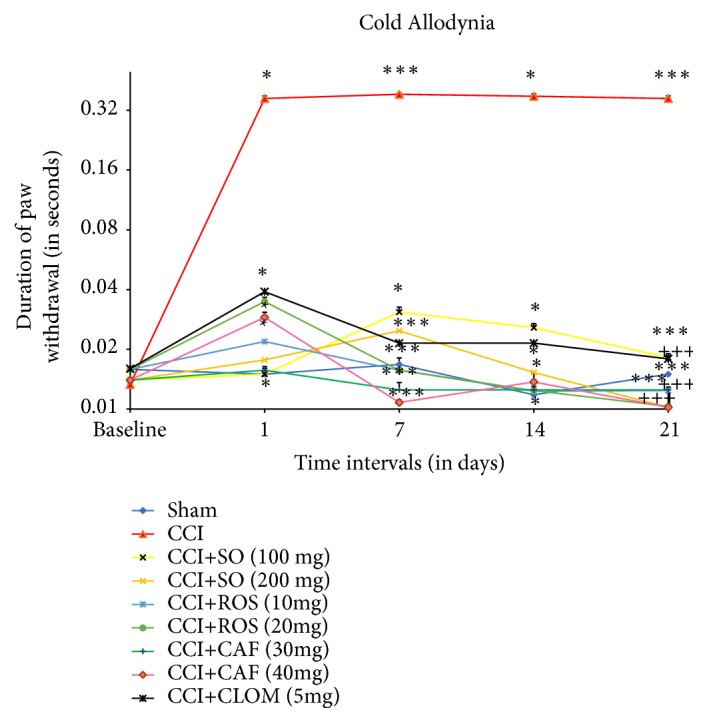
Effect of the administration of* Salvia officinalis *(SO), Rosmarinic acid (ROS), Caffeic acid (CAF), and Clomipramine (CLOM), for 3 weeks, on the paw withdrawal duration (after the acetone drop test) following CCI of the sciatic nerve in mice (n = 6). SO was orally given in doses of 100 and 200 mg/kg. The data are expressed as mean ± SEM. *∗∗∗*P<0.001; *∗*P<0.05 compared with sham group; ^+++^p<0.001 compared with CCI group.

From the 7th day after surgery, the results (Figure 4) indicate that the cold allodynia induced by CCI was significantly attenuated by administration of SO in a dose-dependent manner (100 and 200 mg/kg,* p.o.*), (P< 0.05, vs. CCI group, Figure 4) as analyzed by one-way ANOVA followed by Tukey's multiple comparison test. Rosmarinic acid (10 and 20 mg/kg) reduced hypersensitivity to cold stimulus on day 7 which continued up to day 21 (p< 0.001); the same effect was observed in animals treated with Clomipramine (5 mg/kg). Caffeic acid was also able to significantly decrease paw lifting response, but the most effective and promising effect was observed at the dose (40 mg/kg), (P< 0.001 vs. CCI group).

#### 3.2.3. Effect of* Salvia officinalis* on Thermal Hyperalgesia (Hot-Plate Test)

**Figure 5:**
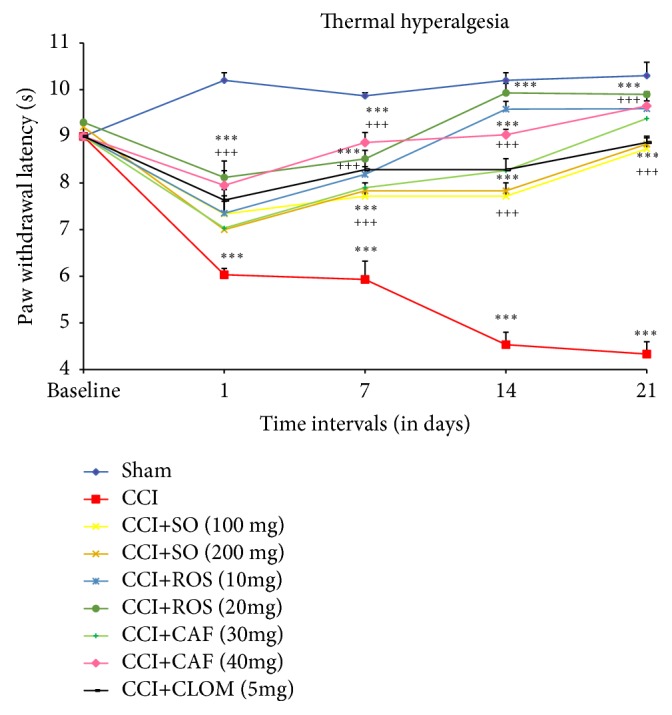
Effect of the administration of* Salvia officinalis, *Rosmarinic acid (ROS), Caffeic acid (CAF), and Clomipramine (CLOM), for 3 weeks, on the hot-plate test latency following CCI of the sciatic nerve in mice (n = 6). SO was orally given in doses of 100 and 200 mg/kg. The data are expressed as mean ± SEM. ^*∗∗∗*^p<0.001 compared with sham group; ^+++^p<0.001 compared with CCI.

Twenty-one days following the CCI of the sciatic nerve, neuropathic pain was induced in all the animals, as evidenced by a significant decrease in hot-plate test latency (Figure 5). Following this, the effect of administration of SO, Rosmarinic, Caffeic acids, and Clomipramine (5 mg/kg) for 3 weeks on hot-plate latency was observed. Administration of SO (100 and 200 mg/kg) attenuated CCI-induced decrease in the nociceptive threshold for thermal hyperalgesia in a dose-dependent manner throughout the study duration after CCI induction,* i.e*., on days 14 and 21; this effect was highly significant as compared with the sham and CCI (p < 0.001) as analyzed by one-way ANOVA followed by Tukey's multiple comparison test, indicating that Rosmarinic acid (10 and 20 mg/kg) dose-dependently promotes heat sensation recovery in CCI mice (Figure 4). As did reference drug Clomipramine (5 mg/kg) Caffeic acid (30 and 40 mg/kg) also produced similar results in opposing CCI-induced hyperalgesia effects (p < 0.001 compared with the CCI group).

#### 3.2.4. Sciatic Nerve Function (SFI)-Walking Test

**Figure 6:**
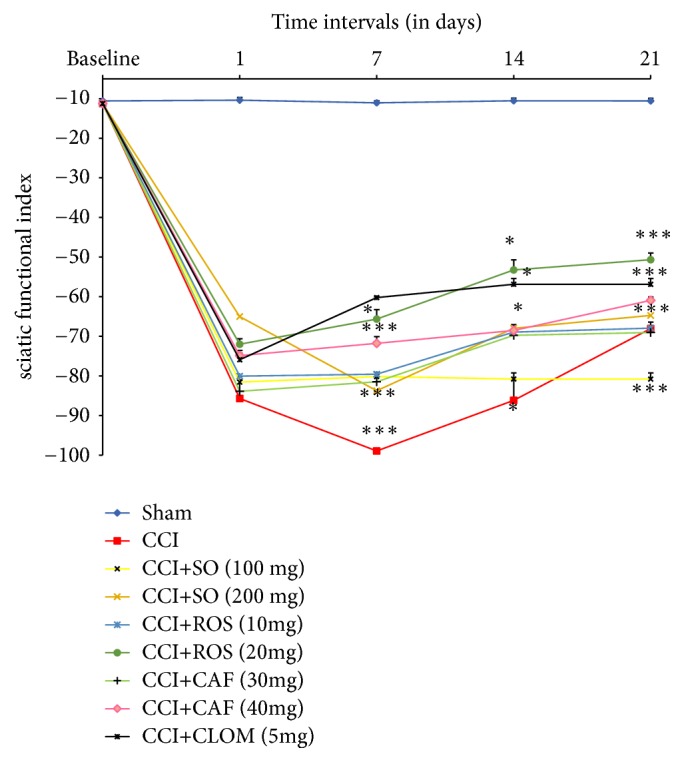
Effect of the administration of* Salvia officinalis *(SO), Rosmarinic acid (ROS), Caffeic acid (CAF), and Clomipramine (CLOM), for 3 weeks on the sciatic functional index in the walking test, following CCI of the sciatic nerve in mice (n = 6). SO was orally given in doses of 100 and 200 mg/kg. The data are expressed as mean ± SEM. *∗*p < 0.05 compared with sham group.

Chronic constriction injury of sciatic nerve resulted in significant development of sciatic functional loss as indicated by a decreased level of SFI when compared to the sham group (Figure 6). One day after surgery, there was no significant difference in SFI among groups (p > 0.05).

Seven days after surgery, SFI was significantly reduced in the mice subjected to CCI (p < 0.05) vs. sham group. The administration of SO in a dose-dependent manner (100 and 200 mg/kg,* p.o.*) attenuated CCI-induced increase in the SFI value compared with the sham group (p < 0.001). Animals treated with Rosmarinic (10 and 20 mg/kg) or Caffeic acids (30 and 40mg/kg) showed significant (p < 0.05) recovery in walking behavior at days 14 and 21 after CCI when compared with group sham group.

Treatment of Clomipramine also produced similar effects over time after surgery.

### 3.3. Hematological and Biochemical Analysis

CCI induced hematologic variations in mice. For instance, we noted a decline in red blood cells (RBC) and Hb count and an upswing in platelets and ESR level (Tables 4 and 5), which were positively amended by SO extract and its major compounds (Rosmarinic and Caffeic acids), together with reference drug Clomipramine.

The levels of WBC, neutrophils, basophils, lymphocytes, and total monocytes increased in the CCI group compared with the sham group (p > 0.05). However, a decrease was observed in the same parameters after the different treatments of CCI mice compared to the CCI group (p > 0.05).

More so, high levels of serum CRP were observed in CCI animals. SO, its phenolic compounds (Rosmarinic and Caffeic acids), and Clomipramine significantly normalized serum CRP levels of CCI mice (Table 6).

### 3.4. Nephrotoxicity and Hepatotoxicity Analysis

As shown in Table 7 chronic treatment with SO (100 and 200 mg/kg,* p.o.*) and Rosmarinic (10 and 20 mg/kg) and Caffeic acids (30 and 40 mg/kg) had no significant effect on plasma levels (Urea, Creatinine, AST, and ALT).

### 3.5. Histopathological Study

**Figure 7:**
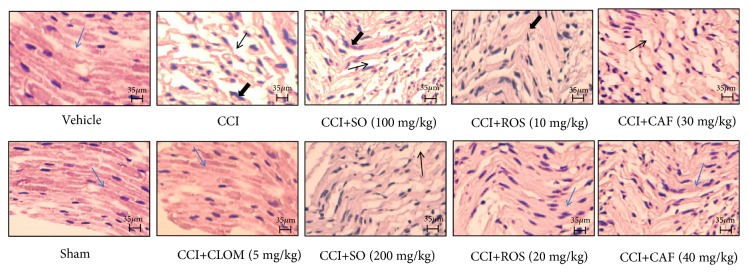
Effect of SO, Rosmarinic acid (ROS), Caffeic acids (CAF), and Clomipramine (CLOM) on sciatic nerve histopathological changes stained with H&E of CCI-induced neuropathy in male mice. Normal mice (vehicle) showed normal fiber arrangement (Blue arrow) and no inflammation. Sham-operated mice showed the same structure as observed in the normal mice; not any marked changes were observed. CCI induced widely separated nerve fibers (thin arrow) and changes in satellite and Schwann cells and showed an increase in the inflammatory cell infiltration (bold arrow). Pretreatment with SO, Rosmarinic and Caffeic acids, and Clomipramine increased axonal regeneration, slightly separated nerve fibers, and normalized the derangement observed in the CCI (H&E 400× and scale bar, 35 *μ*m).

Treatment with ethanolic extract of* Salvia officinalis* (100 and 200 mg/kg,* p.o.*) and Rosmarinic (10 and 20 mg/kg,* i.p.*) and Caffeic acids (30 and 40 mg/kg,* i.p.*) attenuated CCI-induced axonal degeneration and inflammatory changes. More so, CCI mice presented histological alteration as revealed by the marked proliferation of the inflammatory cell and decrease of degenerated fibers nerve fibers (Figure 7).

## 4. Discussion

Peripheral nerve injury like chronic constriction injury (CCI) is frequently used to screen the potential therapeutic agents against neuropathic pain in rodent [34, 43]. It can produce pain-related behaviors similar to those observed in human patients with neuropathy [36]. To the best of our knowledge, the present work is the first study assessing the beneficial effect of chronic treatment with* Salvia officinalis *extract and its major compounds (Rosmarinic and Caffeic acids) in CCI model under standardized experimental conditions. Parameters like mechanical hyperalgesia (von Frey test), paw cold allodynia (acetone test), paw thermal hyperalgesia (hot-plate test), and Sciatic nerve function index (SFI) were evaluated, respectively.

In this study, our data clearly demonstrated behavioral, biochemical (CRP), hematological, and histopathological alternations in CCI animals due to the induction of peripheral neuropathy in mice. These alterations started on the 3rd day after surgery and lasted throughout the experimental period. However, the chronic treatment with SO and Rosmarinic and Caffeic acids suppressed responses to pain behavior, when given daily in dose-dependent manner. The dosage range used during the experiments was tolerable and benign based on the toxicity profile [18, 44, 45]. In addition, the evaluation of nephrotoxicity and hepatotoxicity biomarkers revealed that the different chronic treatments used in this study did not alter Urea, Creatinine, AST, and ALT plasma levels. Therefore, SO and its major compounds seem to be safe regarding liver and renal damage.

The finding of our study showed that chronic administration of SO and its main bioactive components (Rosmarinic and Caffeic acids) significantly increased the sensitivity and reaction time to pain in irritable animals intermediately 7 days after the induction of neuropathic pain and continued for 21 days. Many experimental studies provide evidence of the beneficial effects of SO on acute and chronic models of pain; they are in accordance with our results. It has been shown that hydroalcoholic extract of SO has anti-inflammatory and antinociceptive effects on chemical behavioral models [27, 46]. According to our phytochemical results, SO extract revealed a higher content of flavonoids and phenolic compounds such as Caffeic acid and Rosmarinic acid, which are known for their anti-inflammatory and analgesic activity [47]. Bauer et* al.* reported [48] that SO compounds have analgesic effects via COX_2_ selective inhibitors which have significant inhibitory effects on PGE_2_. Additionally, our results concerning the antineuropathic effect of Rosmarinic acid are in accordance with results reported by Rahbardar and his collaborator in CCI rats [8, 26]. In the existing hematologic variations, it is clear that a rise in ESR and a fall in Hb and RBC count in CCI animals represent inflammation condition [49], which was suppressed by the administration of all the treatments and standard drug for 21 days.

According to the literature and on the basis of our results, the alteration of RBC, Hb, and HCT levels in CCI animals reveals inflammatory anemia. This anemia (called also typically normocytic normochromic anemia) occurred in the setting of acute or chronic inflammation and it manifested by the decrease of RBC parameters. More so, the pathogenesis of neuropathic pain is characterized by neuroinflammation which could be responsible for the decreased RBC, Hb, and HCT parameters observed in our study. The treatment with* Salvia officinalis* and its major compounds increased the RBC level, which could be due to its anti-inflammatory effects [50, 51].

Changes were also observed in WBCs level. More so, the serum concentration of CRP (inflammatory biomarker) is produced as a prompt response to fluctuations in underlying inflammation [52], and CRP synthesis is mainly stimulated by proinflammatory cytokines, such as alpha tumor necrosis (TNF-*α*) [53]. The significantly low levels of serum CRP in CCI animals treated by SO, Rosmarinic and Caffeic acids, and Clomipramine as compared to CCI control mice indicate remission of inflammation. Lately, Rahbardar and his collaborators reported that Rosmarinic acid attenuates the symptoms of neuropathic pain by increasing the levels of TNF-*α* in CCI rats [8]. Moreover, a recent study demonstrates that Caffeic acid relieves neuropathic pain by decreasing the expression of proinflammatory cytokine tumor necrosis factor-*α*, IL-1*β*, and IL-6 [54]. So, the antineuropathic efficacy of* Salvia officinalis* (SO) and its phenolic derivative compounds (Rosmarinic and Caffeic acids) can be attributed to its inhibitory effects on the release of inflammatory mediators.

Interestingly, our results indicate that chronic treatment with SO mimic the effect of Clomipramine (TCA: tricyclic antidepressant) in mice with ligated sciatic nerve. This has only an antiallodynic effect in mechanical test and suppresses the hyperalgesic effect

According to previous studies, tricyclic antidepressants are recommended as one of the first-line treatment in painful neuropathy [55]. Tricyclic antidepressants have a specific analgesic effect and exert their therapeutic effect by recruiting the endogenous opioid system. Their effect can be acutely blocked by a single injection of delta or kappa-opioid receptor antagonist which plays a crucial role in the inhibitory control of pain [56]. Chronic study of treatment with Clomipramine showed antihyperalgesic action in CCI rats and suggested in the implication of the opioid system [57]. However, SO extract (100 and 200 mg/kg) have been observed to have better pharmacotherapeutic effects in comparison to Clomipramine for management of CCI-induced neuropathic pain. Taken together, these data suggest that the endogenous opioid system plays a critical role in chronic TCA relief of neuropathic pain and could also be involved in the beneficial effect of SO extract in CCI model.

An important and new aspect of our study is that SO promotes the motor functional recovery of the injured sciatic nerve and axonal regeneration. This beneficial effect was further evidenced by higher SFI values in CCI mice treated with SO, Rosmarinic and Caffeic acids as well as histopathological analysis. On the basis of data in hand and with support from literature, it is suggested that the beneficial effect of SO and Rosmarinic and Caffeic acids on sciatic nerve regeneration could be attributed to its multiple pharmacological actions like antioxidative, anti-inflammatory, and neuroprotective properties. This has been demonstrated in the current results of this study, although other mechanisms may contribute to the observed neuroprotection. Further studies are needed to investigate the exact mechanism and possible protective effect of* Salvia officinalis* on other forms of neuronal injury.

## 5. Conclusion

In summary, this study demonstrates for the first time that continuous daily administration of SO and its major compounds attenuated neuropathic pain in the chronic constriction injury-induced behavioral changes in mice. Moreover, they possess neuroprotective effects as demonstrated in the results of the walking test. The present study suggests the potential use of* Salvia officinalis* in the treatment of neuropathic pain and the results corroborate the use of this plant in traditional medicine. However, further studies are necessary for the elucidation of its mechanisms of action.

## Figures and Tables

**Table 1:** Total phenolic compounds, total flavonoids compounds, and total tannins of *Salvia officinalis *ethanolic extract.

	TPC (mg GAE/g DM)	TFC (QEeq/1 g DM)	TTC (CAT/ g DM)
SO extract	120.30 ± 0.72	91.12 ± 0.50	22.10± 0.41

All determinations were carried out at least in triplicate and values were averaged and given along the standard error of the mean (mean ± SEM). *Salvia officinalis *(SO), TPC: total phenolic content; TFC: total flavonoid content; TTC: total tannin content.

**Table 2:** Antioxidant activity of *Salvia officinalis *ethanolic extract against DPPH, reducing power, and *β*-carotene methods.

	Extract SO (IC50 mg/ml)	Quercetin (IC50 *μ*g/ml)	BHT (IC50 *μ*g/ml)
DPPH	0.66 ± 0.020	0.10 ± 0.002	0.35 ± 0.002
Reducing power	0.22 ± 0.006	0.07 ± 0.001	0.12 ± 0.008
*β*-carotene -linoleic acid	0.30 ± 0.002	2.62 ± 0.020	0.07 ± 0.001

Values are expressed as mg per ml and represent means of triplicate determination (mean ± SEM).* Salvia officinalis *(SO).

**Table 3:** Quantification of main phenolic compounds in *Salvia officinalis*.

Phenolic compounds	Concentrations (mg EGA/100 g DM)
Gallic acid	13.67
Caffeic acid	15.10
Carnosic acid	13.64
Vanillic acid	17.96
Naringenin	15.84
Epicatechin	14.11
Rosmarinic acid	76.70
Quercetin	28.23
P-Coumaric acid	13.99

**Table 4:** Effect of administration of *Salvia officinalis *extract (SO), Rosmarinic (ROS) and Caffeic acids (CAF), and Clomipramine (CLOM), on hematological parameters in CCI mice. Red blood cells (RBC), hemoglobin (Hb), hematocrit (HCT), mean corpuscular volume (MCV), mean corpuscular hemoglobin (MCH), mean corpuscular hemoglobin concentration (MCHC), platelets, and erythrocyte sedimentation rate (ESR).

Treatment mg/kg	RBC(×10^6^/*μ*)	Hb (g/dL)	HCT (%)	MCV (fl)	MCH (pg)	MCHC (g/dL)	Platelets (×10^3^/*μ*l)	ESR (mm/1st h)
Sham	9.10±0.32	15.23±0.61	51.66±1.22	54.56±0.79	15.35±0.16	30.60±0.70	535.50±4.19	3.46±0.04
CCI	5.83±0.37 *∗∗∗*	10.95±0.55 *∗∗∗*	27.76±0.52 *∗∗∗*	34.91±0.91	16.28±0.42	26.65±0.46	1130.50±2.52 *∗∗∗*	8.00±0.04 *∗∗∗*
CCI + SO 100	6.98±0.21 *∗∗∗*	12.90±0.80 *∗∗∗*	37.70±0.80	43.66±1.21 *∗∗∗*	15.63±0.15	29.16±0.40	869.00±3.41	3.23±0.04
CCI + SO 200	7.83±0.23 +++	13.63±0.26 $\begin{array}{c} {}***,+++ \end{array}$	41.20±0.78 $\begin{array}{c} {}***,+++ \end{array}$	45.41±1.31 +++	16.01±0.20	31.00±0.57 +++	703.50±2.44 +++	3.53±0.05 $\begin{array}{c} {}***,+++ \end{array}$
CCI + ROS 10	8.10±0.21 +++	13.40±0.40	40.41±0.55 *∗∗∗*	46.06±0.92	15.30±0.29	30.33±0.42	675.00±1.23 *∗∗∗*	3.58±0.03 *∗∗∗*
CCI + ROS 20	9.01±0.34 +++	14.70±0.58 $\begin{array}{c} {}***,+++ \end{array}$	44.15±0.77 $\begin{array}{c} {}***,+++ \end{array}$	46.28±1.11 $\begin{array}{c} {}***,+++ \end{array}$	16.21±0.16	32.00±0.44 +++	632.33±1.64 +++	4.08±0.06 +++
CCI + CAF 30	7.76±0.27	13.08±0.65	39.25±0.79	46.05±1.33	15.40±0.21	29.33±0.33	711.66±3.87 *∗∗∗*	4.2±0.03 +++
CCI + CAF 40	7.88±0.28 +++	13.30±0.64 $\begin{array}{c} {}***,+++ \end{array}$	39.90±0.78 $\begin{array}{c} {}***,+++ \end{array}$	45.35±1.03 +++	16.06±0.08	30.66±0.61 +++	691.16±1.99 +++	3.71±0.05 $\begin{array}{c} {}***,+++ \end{array}$
CCI + ClOM 5	8.58±0.20 $\begin{array}{c} {}***,+++ \end{array}$	14.05±0.49 $\begin{array}{c} {}***,+++ \end{array}$	42.15±0.60 $\begin{array}{c} {}***,+++ \end{array}$	46.50±1.51 $\begin{array}{c} {}***,+++ \end{array}$	15.25±0.15	30.66±0.61	623.66±1.40 +++	4.20±0.06 +++

Results are expressed as mean ± SEM (n = 6), using one-way ANOVA followed by Tukey posttest. *∗∗∗*p< 0.001 with sham group; ^+++^p< 0.001 compared with CCI; *Salvia officinalis *(SO), Rosmarinic (ROS), Caffeic acids (CAF), and Clomipramine (CLOM).

**Table 5:** Effect of administration of *Salvia officinalis *extract (SO), Rosmarinic (ROS), Caffeic acids (CAF), and Clomipramine (CLOM), on hematological parameters in CCI mice. White blood cells (WBC), neutrophils (NEUT), eosinophils (EO), basophils (BASO), lymphocytes (LYMPH), and total monocytes (MONO).

Treatment (mg/kg)	WBC (×10^3^/*μ*l)	NEUT (×10^3^/*μ*l)	EO (×10^3^/*μ*l)	BASO (×10^3^/*μ*l)	LYMPH (×10^3^/*μ*l)	MONO (×10^3^/*μ*l)
Sham	2.45±0.01	0.16±0.16	0.00±0.00	0.05±0.01	1.40±0.07	0.04±0.07
CCI	2.56±0.03	0.19±0.19	0.00±0.00	0.21±0.12	1.86±0.21	0.22±0.13
CCI + SO 100	1.83±0.16	0.00±0.00	0.00±0.00	0.07±0.04	2.45±0.07	0.14±0.08
CCI + SO 200	2.72±0.13	0.00±0.00	0.00±0.00	0.07±0.04	1.42±0.06	0.04±0.07
CCI + ROS 10	2.49±0.09	0.00±0.00	0.00±0.00	0.05±0.05	1.43±0.06	0.03±0.04
CCI + ROS 20	2.55±0.07	0.00±0.00	0.00±0.00	0.05±0.01	1.42±0.04	0.03±0.03
CCI + CAF 30	2.52±0.09	0.00±0.00	0.00±0.00	0.05±0.05	1.42±0.04	0.04±0.05
CCI + CAF 40	2.48±0.10	0.00±0.00	0.00±0.00	0.05±0.05	1.44±0.02	0.04±0.05
CCI + ClOM 5	2.56±0.05	0.00±0.00	0.00±0.00	0.06±0.03	1.36±0.01	0.05±0.07

Results are expressed as mean ± SEM (n = 6), using one-way ANOVA followed by Tukey posttest. p> 0.05. *Salvia officinalis *(SO), Rosmarinic (ROS), Caffeic acids (CAF), and Clomipramine (CLOM).

**Table 6:** Effect of administration of *Salvia officinalis *extract and major compounds (Rosmarinic and Caffeic acids) on biochemical parameter in CCI mice (C reactive protein (CRP)).

			Treatments (mg/kg)						
	Sham	CCI	CCI + SO 100	CCI + SO 200	CCI + ROS 10	CCI + ROS 20	CCI + CAF 30	CCI + CAF 40	CCI + CLOM 5
CRP (mg/L)	5.6±0.2	38.0±0.3 +++	12.0±0.3	15.0±0.3 *∗∗∗*, +++	13.5±0.2	15.3±0.3 *∗∗∗*, +++	12.7±0.1	17.1±0.3 *∗∗∗*, +++	11.6±0.2 *∗∗∗*, +++

Results are expressed as mean ± SEM (n = 6), using one-way ANOVA followed by Tukey posttest. *∗∗∗*p < 0.001 with sham group; ^+++^p <0.001 compared with CCI. *Salvia officinalis *(SO), Rosmarinic (ROS), Caffeic acids (CAF), and Clomipramine (CLOM).

**Table 7:** Effect of administration of *Salvia officinalis *extract and major compounds (Rosmarinic and Caffeic acids) on biochemical parameters in CCI mice. Urea, Creatinine, Aspartate Aminotransferase (AST), and Alanine Aminotransferase (ALT).

				Treatments (mg/kg)					
	Sham	CCI	CCI + SO 100	CCI + SO 200	CCI + ROS 10	CCI + ROS 20	CCI + CAF 30	CCI + CAF 40	CCI + CLOM 5
Urea (g/l)	0.20± 0.01	0.20± 0.01	0.20±0.02	0.30± 0.02	0.20± 0.01	0.30±0.01	0.20± 0.02	0.30±0.02	0.30±0.01
Creatinine (mg/l)	1.40±0.10	1.30±0.00	1.50± 0.10	1.40± 0.05	1.50±0.10	1.50± 0.10	1.50± 0.10	1.60±0.10	1.60±0.10
ALT (U/L)	48.20±1.40	51.70± 1.40	55.00± 0.90	50.30± 1.10	54.30± 1.10	52.40±1.30	49.80± 0.90	49.00±0.90	50.00±0.90
AST (U/L)	165.00 ±2.10	164.40± 1.20	164.80± 0.90	179.10± 1.30	164.80±0.90	165.20±1.30	170.00±1.30	166.40±1.10	171.00±1.30

Results are expressed as mean ± SEM (n = 6), using one-way ANOVA followed by Tukey posttest. p> 0.05. *Salvia officinalis *(SO), Rosmarinic (ROS), Caffeic acids (CAF), and Clomipramine (CLOM).

## Data Availability

The data used to support the findings of this study are available from the corresponding author upon request.

## Abbreviations

SO: * Salvia officinalis*
CCI: Chronic constriction injury
TPC: Total phenolic content
TFC: Total flavonoid content
TTC: Total tannin content
ROS: Rosmarinic acid
CAF: Caffeic acid
CLOM: Clomipramine
WBC: White blood cell
RBC: Red blood cell
Hb: Hemoglobin
ESR: Erythrocyte sedimentation rate
CRP: C reactive protein
AST: Aspartate transaminase
ALT: Alanine transaminase
TCAs: Clomipramine a tricyclic antidepressants
DPPH: 2,2-Diphenyl-1-picrylhydrazyl
BHT: Butylated hydroxyl toluene
HPLC: High-performance liquid chromatography
SFI: Sciatic nerve function index.
